# Time-dependent risk of sleep disorders in patients with epilepsy: a nationwide cohort study

**DOI:** 10.1186/s12883-026-05038-6

**Published:** 2026-06-10

**Authors:** Seung Won Lee, Chaeyoon Kang, Unbi Choi, Hohyun Jung, Youngoh Bae

**Affiliations:** 1 Department of Precision Medicine, Sungkyunkwan University School of Medicine, Suwon, 16419 Republic of Korea; 2 Personalized Cancer Immunotherapy Research Center, Sungkyunkwan University School of Medicine, Suwon, 16419 Republic of Korea; 3 Department of Artificial Intelligence, Sungkyunkwan University, Suwon, 16419 Republic of Korea; 4 Department of Family Medicine, Kangbuk Samsung Hospital, Sungkyunkwan University School of Medicine, Seoul, 03181 Republic of Korea; 5 Sungkyunkwan University School of Medicine, Seoul, 06355 Republic of Korea; 6 Department of Statistics, Sungshin Women’s University, Seoul, 02844 Republic of Korea; 7 Data Science Center, Sungshin Women’s University, Seoul, 02844 Republic of Korea; 8 Department of Neurological Surgery, Asan Medical Center, University of Ulsan College of Medicine, Seoul, 05505 Republic of Korea

**Keywords:** Epilepsy, Sleep disorder, Time-stratified analysis, Risk factor

## Abstract

**Background:**

Sleep disturbances are common yet underrecognized comorbidities in epilepsy, adversely affecting seizure control and quality of life. This study aimed to evaluate the long-term risk and risk factors of sleep disorders among patients with newly diagnosed epilepsy using a nationwide cohort with 10 years of follow-up.

**Methods:**

Data were obtained from the Korean National Health Insurance Service (2002–2013). Newly diagnosed patients with epilepsy (ICD-10 codes G40–G41; *n* = 2,414) were matched by age and sex to controls (*n* = 24,140) without a prior history of sleep disorders (ICD-10 codes F51, G47). The primary outcome was the incidence of newly diagnosed sleep disorders. Incidence rates (IRs), incidence rate ratios (IRRs), and adjusted hazard ratios (aHRs) were calculated using multivariable and time-stratified Cox regression models.

**Results:**

During follow-up, 15.0% of patients with epilepsy and 8.1% of controls developed sleep disorders, corresponding to IRs of 41.1 and 20.1 per 1,000 person-years (IRR = 2.05, 95% CI = 1.83–2.29). The risk was highest within the first two years after epilepsy diagnosis (aHR = 2.48, 95% CI = 1.93–3.11) and declined thereafter. Younger patients (< 60 years), men, and former smokers exhibited higher risks.

**Conclusions:**

Patients with epilepsy were associated with an increased risk of developing sleep disorders, particularly in the early post-diagnosis period and among younger male patients and former smokers. These findings emphasize the need for early screening and management. Future studies incorporating medication profiles and diverse populations may further clarify these associations.

**Supplementary Information:**

The online version contains supplementary material available at 10.1186/s12883-026-05038-6.

## Background

Epilepsy is a neurological disorder characterized by recurrent seizures that are induced by the transient abnormal excitation of brain cells [1]. It is a chronic neurological condition with an annual incidence of approximately 61.4 cases per 100,000 individuals, contributing significantly to the global disease burden [2]. Beyond seizures, epilepsy frequently coexists with various sleep disorders—including insomnia, excessive daytime sleepiness, and sleep apnea—that can negatively affect seizure control, cognitive function, and quality of life [3, 4].

Sleep is a physiological process essential for brain and body function recovery, playing a crucial role in maintaining mental and physical health [5]. The interaction between epilepsy and sleep is bidirectional and neurobiologically grounded: abnormal cortical excitability in epilepsy can disrupt sleep–wake homeostasis, while alterations in sleep architecture—especially reductions in slow-wave or REM sleep—can facilitate epileptic discharges and lower the seizure threshold [6–9]. Neurotransmitter dysregulation involving γ-aminobutyric acid and orexin pathways further links sleep instability with epileptic activity [10, 11]. Patients with epilepsy with coexisting sleep disorders may experience greater challenges in seizure control [12].

Sleep disturbances adversely affect both the physical and mental health of patients and significantly diminish their quality of life. Understanding the bidirectional interaction between epilepsy and sleep disorders is therefore essential for optimizing clinical management. Several studies have demonstrated a higher prevalence of sleep disorders among patients with epilepsy than among the general population, although estimates vary considerably across studies. The prevalence of insomnia among individuals with epilepsy was reported to be 14.5%, more than three times higher than that in the general population [13], while the prevalence of excessive daytime sleepiness was 11.1%, more than twice that of the general population [14]. However, studies on the prevalence of sleep disorders in patients with epilepsy have shown inconsistent results [15], with reported insomnia rates ranging from 36.0% to 74.4% and obstructive sleep apnea rates ranging from 7.7% to 75.7% [16].

These discrepancies are likely due to variations in study methodologies and small sample sizes. Most prior investigations were hospital-based and cross-sectional, which limits generalizability and hinders evaluation of long-term patterns. Consequently, the temporal evolution of sleep disorders following an epilepsy diagnosis remains poorly understood at the population level.

This study aims to investigate the long-term risk and time-varying patterns of sleep disorders in patients with newly diagnosed epilepsy using a large-scale, nationwide cohort with 10 years of follow-up. By leveraging data from the Korean National Health Insurance Service (KNHIS)—which covers nearly the entire Korean population—this research provides a comprehensive assessment of sleep disorder incidence and its risk factors in an unselected, population-based cohort. The findings are expected to enhance understanding of the dynamic relationship between epilepsy and sleep disorders and to offer novel evidence supporting early detection and management strategies that improve patient outcomes.

## Methods

### Study design and data sources

This was a retrospective, population-based cohort study using the KNHIS claims database and the National Health Screening Program (NHSP) data from 2002 to 2013. South Korea’s National Health Insurance system is a universal health insurance program that covers approximately 98% of the population and includes diagnostic information using the International Classification of Diseases, 10th Revision (ICD-10) codes, healthcare use records, prescription data, and hospitalization details. Additionally, the NHSP data provide health examination results and lifestyle information, including smoking status, body mass index (BMI), blood pressure, blood glucose levels, and total cholesterol levels. Single imputation using predictive mean matching was employed in cases of missing data. This study was approved by the Institutional Review Board of Sungshin Women’s University (SSWUIRB-2025-046). As the data were deidentified prior to use, the requirement for informed consent was waived. This study was conducted in accordance with the Code of Ethics of the World Medical Association (Declaration of Helsinki).

### Participant selection

The study population initially comprised adults who underwent a health examination between January 2002 and December 2003. The case group was defined as individuals diagnosed with epilepsy (ICD-10 codes G40 and G41) at least twice between 2004 and 2013 (Supplementary Table S1), to minimize potential diagnostic miscoding or transient seizure events. The date of the second diagnosis was established as the index date. A washout period from 2002 to 2003 was applied to include only newly diagnosed cases, and individuals diagnosed with epilepsy during this period were excluded from the study. The following exclusion criteria were applied: (1) absence of health examination records within two years prior to the index date, (2) aged < 20 year or > 80 years at the index date, and (3) a prior diagnosis of sleep disorders (ICD-10 codes F51 and G47) within two years before the index date.

The control group was selected from individuals who participated in the NHSP between 2004 and 2013 and had no history of epilepsy. Controls were randomly selected at a 1:10 ratio and matched to cases based on sex, age, and year of health examination. The index date for the controls was set to match that of the corresponding case.

### Study outcome

The primary outcome of this study was the incidence of newly diagnosed sleep disorders (ICD-10 codes F51 and G47), as shown in Supplementary Table S1. To enhance clinical interpretability, sleep disorders were further categorized as follows:

- *F51*: nonorganic sleep disorders (e.g., insomnia, nonorganic hypersomnia, sleep–wake schedule disorders, sleepwalking, night terrors), and.
- *G47*: organic sleep disorders (e.g., sleep apnea, narcolepsy, hypersomnia, circadian rhythm sleep disorder, and other sleep disturbances).

The event date was defined as the date of the first recorded sleep disorder diagnosis after the index date. Individuals without a sleep disorder diagnosis during follow-up were considered event-free until censoring or study endpoint.

Sleep disorders were defined based on ICD-10 codes, and the occurrence of an event was determined by the date of the first sleep disorder diagnosis after the index date. Those who did not receive a sleep disorder diagnosis during the follow-up period were considered event-free until the endpoint of this study.

### Baseline characteristics

Baseline characteristics and covariates of the study population included sex, age, income level, BMI, smoking status, blood pressure (both systolic and diastolic), total cholesterol, and fasting blood sugar. Age was categorized into six groups in 10-year intervals, starting from 20 years. The BMI was classified into underweight (< 18.5 kg/m^2^), normal weight (18.5–24.9 kg/m^2^), and overweight (≥ 25 kg/m^2^) categories. Blood pressure was classified using systolic blood pressure cutoffs of 120 mmHg and 140 mmHg, and diastolic blood pressure cutoffs of 80 mmHg and 90 mmHg. Fasting blood sugar was categorized using 100 mg/dL and 126 mg/dL as the cutoff values. Income levels were stratified into high and low groups based on the median income.

### Statistical analysis

Standardized differences were used to compare the baseline characteristics between the case and control groups. The crude incidence rate (IR) per 1,000 person-years and the incidence rate ratio (IRR) were calculated to compare the incidence of sleep disorders between groups. To assess the association between epilepsy and the risk of sleep disorder incidence, a multivariable Cox proportional hazards regression model was applied, adjusting for sex, age, income level, smoking status, and BMI. Covariates were selected based on their established relevance to both epilepsy and sleep disorders and the availability of reliable data from the National Health Screening Program. Sex, age, income level, smoking status, and BMI were included to account for demographic, socioeconomic, behavioral, and metabolic factors known to influence sleep quality and seizure outcomes. Blood pressure was excluded to avoid multicollinearity with BMI and because its relationship with sleep disorders is considered secondary or mediated through other metabolic pathways rather than causal.

Adjusted hazard ratios (aHRs) with 95% confidence intervals (CIs) were reported.

Schoenfeld’s residual analysis was conducted to evaluate the proportional hazards assumption. As the assumption was violated, the 10-year follow-up period was divided into five two-year intervals, allowing time-stratified extended Cox regression analysis. This approach enabled examination of how the relative risk of sleep disorders evolved across clinically meaningful time windows after epilepsy diagnosis. Subgroup analyses were further conducted by sex (men vs. women) and age (< 60 vs. ≥60 years). All statistical analyses were performed using R version 4.4.1, and two-sided *p* values < 0.05 were considered statistically significant.

## Results

**Fig. 1:**
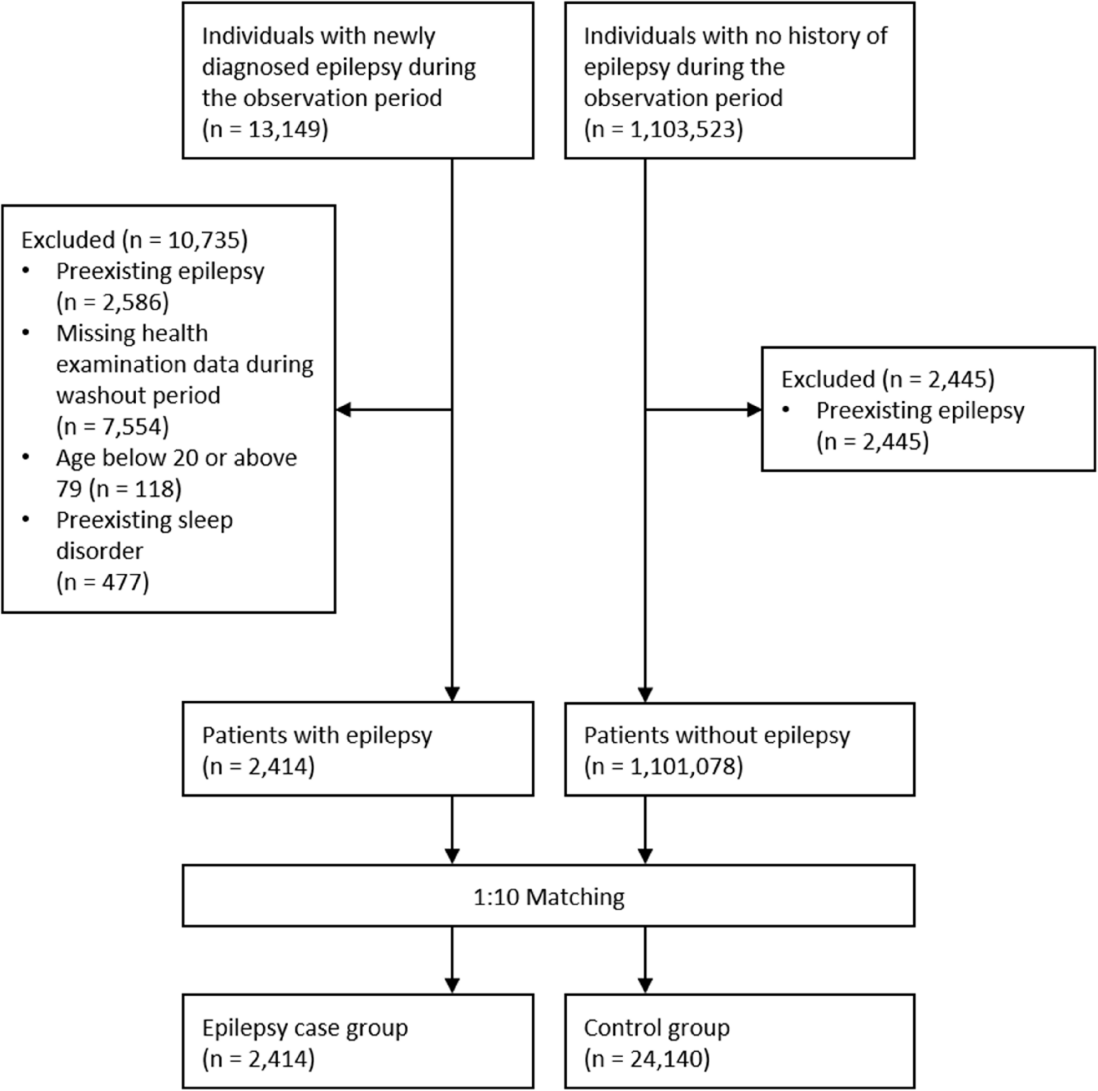
Flowchart for epilepsy case and control group selection

Among the 1,113,656 individuals aged 20 years or older in the KNHIS cohort, 2,414 epilepsy cases and 24,140 matched controls (matched by age, sex, and health examination year) were included after applying the exclusion criteria (Fig. 1). The mean follow-up duration was 4.0 years (standard deviation [SD] = 2.7) for the epilepsy group and 3.7 years (SD = 2.7) for the control group. Table 1 presents the demographic characteristics of the study population. Among all participants, 53.7% (*n* = 1,297) were men, and 54.7% (*n* = 1,321) were aged younger than 60 years. The standardized differences for all demographic and clinical variables between the epilepsy and control groups were below 0.1, indicating negligible differences.

**Table 1:** Demographic and clinical characteristics of patients with epilepsy and the control group

		Patients with epilepsy (*n* = 2,414)	Control group (*n* = 24,140)	Standardized difference
Sex	Male	1,297 (53.7)	12,970 (53.7)	0
	Female	1,117 (46.3)	11,170 (46.3)	
Age (years)	20–29	89 (3.7)	890 (3.7)	0
	30–39	183 (7.6)	1,830 (7.6)	
	40–49	441 (18.3)	4,410 (18.3)	
	50–59	608 (25.2)	6,080 (25.2)	
	60–69	658 (27.3)	6,580 (27.3)	
	≥ 70	435 (18.0)	4,350 (18.0)	
Smoking status	Yes	463 (19.2)	4,823 (20.0)	0.03
	No	1,560 (64.6)	15,433 (63.9)	
	Ex-smoker	267 (11.1)	2,700 (11.2)	
	Missing	124 (5.1)	1,184 (4.9)	
BMI (kg/m^2^)	< 18.5	89 (3.7)	743 (3.1)	0.08
	18.5 to < 25	1,465 (60.7)	15,288 (63.3)	
	≥ 25	856 (35.5)	8,101 (33.6)	
Height (mean, SD)		161.54 (8.81)	161.74 (8.99)	0.02
Weight (mean, SD)		62.58 (10.81)	62.69 (10.70)	0.01
Total cholesterol (mg/dL)	< 200	1,415 (58.6)	13,596 (56.3)	0.07
	≥ 200	996 (41.3)	10,530 (43.6)	
Systolic blood pressure (mm Hg)	< 120	782 (32.4)	7,788 (32.3)	0.02
	120 to < 140	1,163 (48.2)	11,809 (48.9)	
	≥ 140	468 (19.4)	4,534 (18.8)	
Diastolic blood pressure (mm Hg)	< 80	1,164 (48.2)	11,857 (49.1)	0.03
	80 to < 90	894 (37.0)	8,685 (36.0)	
	≥ 90	355 (14.7)	3,590 (14.9)	
FBS (mg/dL)	< 100	1550 (64.2)	15,616 (64.7)	0.03
	100 to < 126	637 (26.4)	6,451 (26.7)	
	≥ 126	225 (9.3)	2,067 (8.6)	
Income	Low	942 (39.0)	9,062 (37.5)	0.04
	High	1,472 (61.0)	15,078 (62.5)	

*Abbreviations*: *BMI* Body mass index, *FBS* Fasting blood sugar

Table 2 presents the crude IRs and risk ratios for sleep disorders in the epilepsy and control groups. During the follow-up period, 362 individuals (15.0%) in the epilepsy group and 1,956 individuals (8.1%) in the control group were diagnosed with sleep disorders. The crude IR per 1,000 person-years was 41.07 (95% CI, 36.87–45.38) in the epilepsy group and 20.06 (95% CI, 19.18–20.95) in the control group, yielding an IRR of 2.05 (95% CI, 1.83–2.29). In the sex-stratified analysis, the crude IR of sleep disorders was lower in male patients with epilepsy (39.84 [95% CI, 34.18–45.50]) than in female patients (42.52 [95% CI, 36.34–48.95]). However, the IRR was higher in men (2.37 [95% CI, 2.03–2.77]) than in women (1.76 [95% CI, 1.50–2.07]). A similar pattern was observed in the age-stratified analysis, where patients younger than 60 years had a lower crude IR (33.24 [95% CI, 28.35–38.31]) than those aged 60 years or older (53.04 [95% CI, 45.58–60.78]); however, the former exhibited a higher IRR. When further stratified by both sex and age, the highest IRR was observed in men aged younger than 60 years (3.44 [95% CI, 2.73–4.32]), followed by women aged younger than 60 years (2.00 [95% CI, 1.58–2.54]), men aged 60 years or older (1.83 [95% CI, 1.47–2.27]), and women aged 60 years or older (1.59 [95% CI, 1.28–1.99]). When stratified by smoking status, current (2.42 [95% CI, 1.85–3.18]) and former (4.26 [95% CI, 2.98–6.09]) smokers had a higher IRR than non-smokers (1.79 [95% CI, 1.56–2.05]). By contrast, analyses based on income level, BMI, and total cholesterol showed only minor differences between the groups.

**Table 2:** Crude incidence rates and risk ratios for sleep disorder among patients with epilepsy and controls

		Patients with epilepsy (*n* = 2,414)			Control group (*n* = 24,140)			IRR (95% CI)
		Cases	Person-years	IR per 1,000 person-years (95% CI)	Cases	Person-years	IR per 1,000 person-years (95% CI)	
All		362	8,813.76	41.07 (36.87–45.38)	1,956	97,507.63	20.06 (19.18–20.95)	2.05 (1.83–2.29)
Sex	Male	190	47,68.95	39.84 (34.18–45.50)	912	54,207.36	16.82 (15.74–17.93)	2.37 (2.03–2.77)
	Female	172	4,044.82	42.52 (36.34–48.95)	1,044	43,300.26	24.11 (22.66–25.59)	1.76 (1.50–2.07)
Age (years)	< 60	177	5,325.57	33.24 (28.35–38.31)	748	58,562.70	12.77 (11.87–13.69)	2.60 (2.21–3.07)
	≥ 60	185	3,488.19	53.04 (45.58–60.78)	1,208	38,944.93	31.02 (29.27–32.79)	1.71 (1.46–2.00)
Sex and age (years)	Male, < 60	95	2,966.50	32.02 (25.62–38.77)	311	33,372.27	9.32 (8.30–10.37)	3.44 (2.73–4.32)
	Male, ≥ 60	95	1,802.44	52.71 (42.17–63.80)	601	20,835.09	28.85 (26.54–31.20)	1.83 (1.47–2.27)
	Female, < 60	82	2,359.07	34.76 (27.55–42.39)	437	25,190.43	17.35 (15.76–18.98)	2.00 (1.58–2.54)
	Female, ≥ 60	90	1,685.75	53.39 (42.71–64.66)	607	18,109.84	33.52 (30.87–36.22)	1.59 (1.28–1.99)
Income	Low	139	3,412.77	40.73 (33.99–47.76)	700	36,594.05	19.13 (17.74–20.55)	2.13 (1.77–2.55)
	High	223	5,401.00	41.29 (35.92–46.84)	1,256	60,913.57	20.62 (19.49–21.77)	2.00 (1.74–2.31)
Smoking status	Yes	63	1,708.70	36.87 (28.09–46.23)	311	20,438.82	15.22 (13.55–16.93)	2.42 (1.85–3.18)
	No	235	5,800.00	40.52 (35.34–45.86)	1,408	62,082.47	22.68 (21.50–23.87)	1.79 (1.56–2.05)
	Ex-smoker	41	658.45	62.27 (44.04–82.01)	113	7,734.88	14.61 (12.02–17.32)	4.26 (2.98–6.09)
BMI (kg/m^2^)	< 18.5	14	275.18	50.88 (25.44–79.95)	65	2,945.13	22.07 (16.98–27.50)	2.31 (1.29–4.11)
	18.5 to < 25	224	5,489.40	40.81 (35.52–46.27)	1,226	62,037.20	19.76 (18.67–20.87)	2.06 (1.79–2.38)
	≥ 25	123	3,034.44	40.53 (33.61–47.78)	664	32,487.63	20.44 (18.90–22.01)	1.98 (1.64–2.40)
Total cholesterol (mg/dL)	< 200	207	5,110.80	40.50 (35.02–46.18)	1,042	54,973.42	18.95 (17.81–20.12)	2.14 (1.84–2.48)
	≥ 200	154	3,695.62	41.67 (35.18–48.44)	912	42,444.99	21.49 (20.10–22.90)	1.94 (1.63–2.30)

*Abbreviations*: *IR *Incidence rate, *CI *Confidence interval, *BM I*Body mass index

**Fig. 2:**
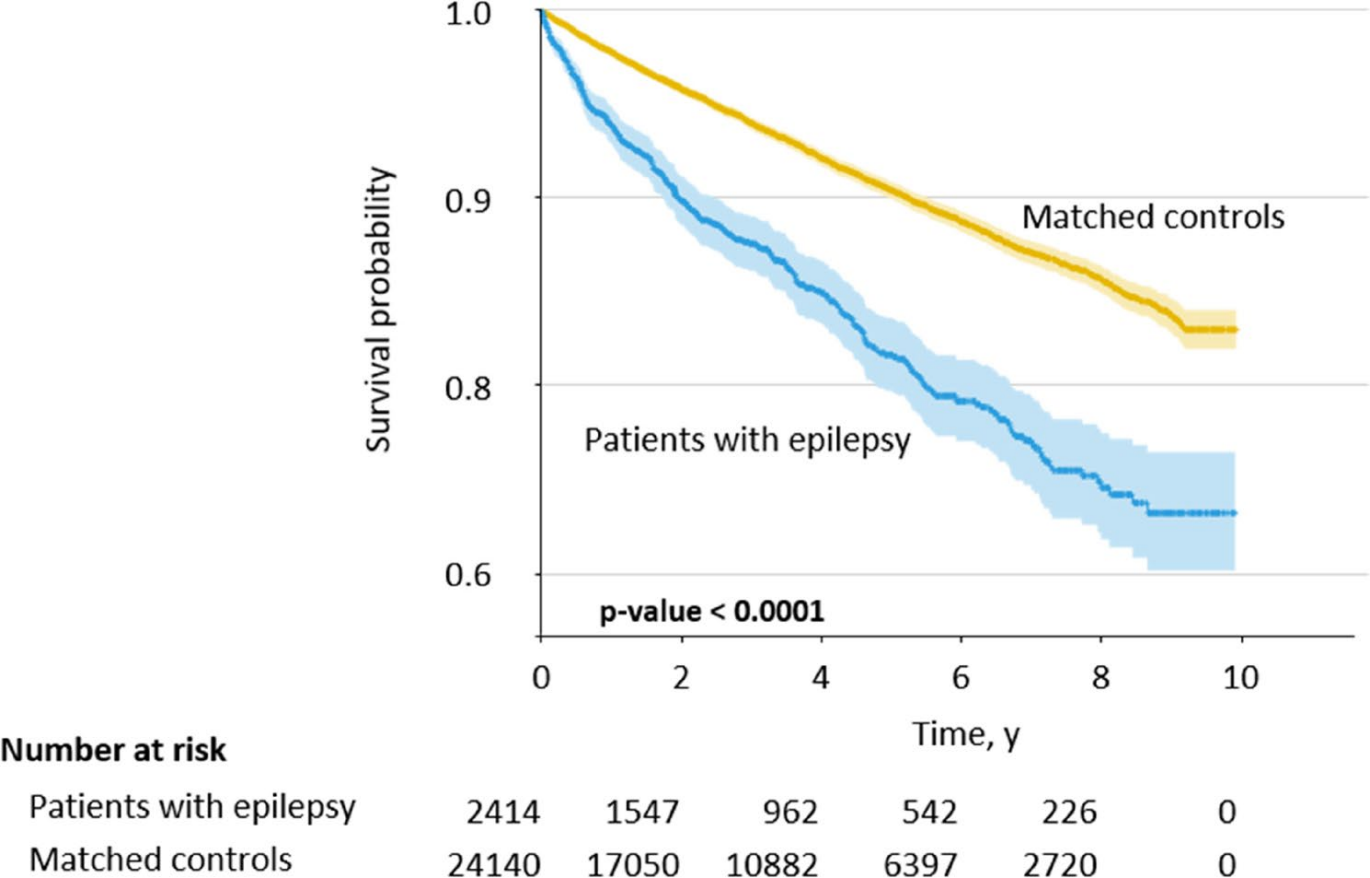
Kaplan–Meier curve for survival probability from sleep disorders in patients with epilepsy and matched controls

**Fig. 3:**
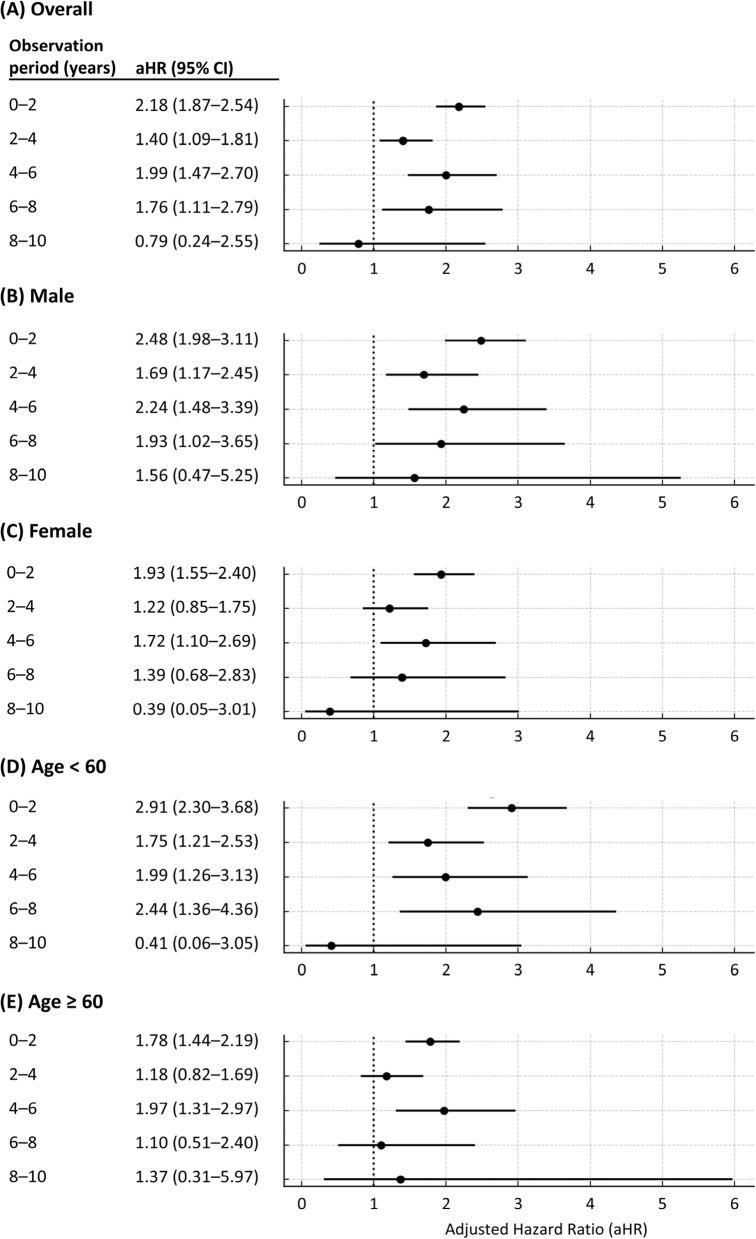
Forest plots illustrating the risk of sleep disorder among all patients with epilepsy (A), stratified by sex (B, C: men, women) and by age group (D, E: < 60 years, ≥ 60 years) using an extended time-stratified Cox proportional hazards model

The Kaplan–Meier survival curve illustrating the cumulative survival probability for sleep disorder incidence in the epilepsy and control groups is presented in Fig. 2. Unlike the control group, which exhibited a relatively steady slope throughout the follow-up period, the epilepsy group showed a steep initial decline in survival probability shortly after diagnosis. This pattern indicates a violation of the proportional hazards assumption, as confirmed by the analysis of scaled Schoenfeld residuals. The extended time-stratified Cox regression model was applied to address this violation, dividing the 10-year follow-up period into 2-year intervals. The results of this time-stratified analysis are illustrated in Fig. 3, demonstrating how the risk of sleep disorder incidence changes over time among patients with epilepsy.

The time-stratified Cox regression analysis indicated that the risk of developing sleep disorders remained elevated for approximately 8 years following an epilepsy diagnosis but did not increase significantly during the 8–10-year period. Throughout the initial eight years, the risk fluctuated but remained approximately twofold compared to the control group. Among the time intervals, the lowest risk was observed in the two- to four-year period, with an aHR of 1.40 (95% CI, 1.09–1.81). By contrast, the highest risk was observed during the four- to six-year period, with an aHR of 1.99 (95% CI, 1.47–2.20).

## Discussion

In this nationwide, large-scale cohort study with a 10-year follow-up, which included 2,414 patients with epilepsy and 24,140 matched controls based on sex and age, we found that patients with epilepsy was associated with risk of sleep disorders compared to the control group. Over the 10-year follow-up period, the association between epilepsy and sleep disorders corresponded to an approximately twofold higher risk compared to the control groupIn the subgroup analysis by sex and age, the increase in risk was most evident among men and those younger than 60 years. The time-stratified analysis revealed that the association between epilepsy and sleep disorders was strongest during the early period (zero to two years) after diagnosis and gradually weakened thereafter.

A comparison of sleep disorder IRs between the epilepsy and control groups showed that patients with epilepsy had an IRR of 2.05 (95% CI, 1.83–2.29; Table 2). Similar trends have been reported in previous studies. A retrospective cohort study using Taiwan’s national health insurance data analyzed over 500,000 individuals and reported an aHR of 2.37 (95% CI, 1.47–3.82) for non-organic sleep disorders, consistent with our findings [17]. Similarly, a large-scale survey conducted in the United States involving 3,488 patients with epilepsy reported a prevalence ratio of 1.44 (95% CI, 1.29–1.60) for sleep disorders and sleep apnea, aligning with the results of our study [18]. By contrast, a German retrospective cohort study comparing 7,942 patients with epilepsy and matched controls indicated no significant association between epilepsy and sleep disorders, reporting an odds ratio of 0.95 (95% CI, 0.84–1.08) [19]. This discrepancy may be due to differences in sleep disorder classification, as the German study did not include non-organic sleep disorders, whereas our study considered both non-organic and organic sleep disorders.

Epilepsy and sleep are closely interconnected, with multiple studies highlighting their neurophysiological relationship [20]. Non-rapid eye movement sleep facilitates the occurrence and propagation of seizures and interictal epileptic discharges, whereas rapid eye movement sleep tends to suppress them. Simultaneously, seizures can disrupt the sleep cycle, leading to disturbances in sleep homeostasis. Abnormal neuronal hyperexcitability and increased synaptic currents can interfere with sleep regulation, leading to disruptions in sleep [21]. Additionally, postictal alterations in melatonin receptor function and dysregulation of neurotransmitters such as orexin can destabilize the sleep–wake cycle, making normal sleep transitions more difficult [22, 23]. Moreover, dysfunction of the γ-aminobutyric acid system—a key inhibitory mechanism regulating brain excitability—has been implicated in changes to sleep architecture [24, 25]. These seizure-related effects contribute to reduced sleep stability, prolonged sleep latency, and fragmented sleep stages, ultimately resulting in poor overall sleep quality [26, 27].

In addition, anti-seizure medications (ASMs) can directly influence sleep architecture. Medications such as carbamazepine, levetiracetam, phenytoin, and phenobarbital alter sleep structure by inducing sedation or promoting wakefulness [28]. In particular, daytime sleepiness and impaired sleep continuity caused by ASMs may contribute to or exacerbate sleep disorders [29].

To summarize, the increased risk of sleep disorders in patients with epilepsy can be attributed to a complex interplay of neurobiological and pharmacological factors, including neuronal hyperexcitability, dysregulation of neurotransmitters, dysfunction of the γ-aminobutyric acid system, and the effects of ASMs.

The IR of sleep disorders per 1,000 person-years in female patients with epilepsy was slightly higher than in male patients (42.52 [95% CI, 36.34–48.95] vs. 39.84 [95% CI, 34.18–45.50]). However, the IRR, which represents the relative risk increase compared to the control group, was higher in men (2.37 [95% CI, 2.03–2.77] vs. 1.76 [95% CI, 1.50–2.07] for women). Similarly, in the aHR analysis, male patients with epilepsy had a higher risk of sleep disorders during the early period (zero to two years) following diagnosis, with an aHR of 2.48 (95% CI, 1.93–3.11), compared to 1.93 (95% CI, 1.55–2.40) in female patients. This suggests that male patients faced a relatively higher risk of sleep disorders shortly after their diagnosis. Previous studies have also reported sex differences in sleep quality among patients with epilepsy. A study involving 220 patients with epilepsy assessed sleep quality using the Pittsburgh Sleep Quality Index and found that male patients exhibited poorer overall sleep quality than female patients [30]. A possible explanation for these findings is that male patients may experience higher stress and anxiety. A cross-sectional population-based study drawing on data from the UK General Practice Research Database indicated that men had a higher prevalence of depression than women (2.59 [95% CI, 2.35–2.86] vs. 1.79 [95% CI, 1.67–1.93]) and anxiety disorders (2.56 [95% CI, 2.25–2.90] vs. 1.95 [95% CI, 1.77–2.16]) [31]. The social and economic stress associated with epilepsy, particularly the financial burden often borne by men, may have a greater psychological impact on men, potentially contributing to the development of sleep disorders such as insomnia and sleep apnea.

In the age-stratified IRR analysis, patients with epilepsy younger than 60 years had a higher IRR for sleep disorders (2.60 [95% CI, 2.21–3.07]) than those aged 60 years or older (1.71 [95% CI, 1.46–2.00]). Since the crude IR of sleep disorders was higher in older adults, this finding is crucial. A possible explanation is that the psychosocial impact of epilepsy is more pronounced in younger individuals, rendering them more vulnerable to sleep disturbances. The social stigma associated with epilepsy is stronger in younger age groups, potentially leading to increased psychological stress and a higher risk of sleep disorders [32]. Younger patients with epilepsy may experience greater disadvantages in education, employment, and social relationships, amplifying psychosocial stress and contributing to the higher IRR of sleep disorders observed in this group.

In the analysis by smoking status, former smokers had the highest IRR for sleep disorders (4.26 [95% CI, 2.98–6.09]), followed by current smokers (2.42 [95% CI, 1.85–3.18]) and non-smokers (1.79 [95% CI, 1.56–2.05]). Smoking has been associated with poor sleep quality and increased seizure susceptibility. Previous studies have reported links between smoking and delayed sleep onset, difficulty waking, and excessive daytime sleepiness [33]. A dose–response relationship has also been observed between smoking intensity and sleep disturbances [34], likely due to the stimulatory effects of nicotine on the central nervous system, which can interfere with normal sleep regulation [35]. In patients with epilepsy, smoking may further contribute to seizure susceptibility. A study involving 92 patients with epilepsy found that current smokers had a 3.6-fold higher risk of experiencing seizures during the previous year compared with non-smokers [36], and another study of 524 patients reported that current smokers exhibited significantly higher seizure frequency than non-smokers [37]. These findings suggest that smoking and sleep disturbances are interrelated through both neurophysiological and behavioural mechanisms. In the short term, nicotine withdrawal after smoking cessation may lead to anxiety, insomnia, and altered sleep architecture, whereas chronic nicotine exposure may induce long-term changes in arousal regulation and neurotransmitter balance. This dual effect could explain the higher IRR observed among both former and current smokers. However, because smoking cessation and its withdrawal symptoms can precede the diagnosis of sleep disorders, reverse causality cannot be ruled out. Therefore, the relationship between smoking behaviour, seizure activity, and sleep disorders should be interpreted as associative rather than causal.

Moreover, the IR of sleep disorders per 1,000 person-years was highest among former smokers (62.27 [95% CI, 44.04–82.01]), followed by non-smokers (40.52 [95% CI, 35.34–45.86]) and current smokers (36.87 [95% CI, 28.09–46.23]). The higher incidence of sleep disorders in former smokers compared to current smokers may be attributed to the withdrawal effects of smoking cessation. Symptoms such as anxiety, depression, and insomnia, which peak within the first week of quitting and can persist for up to a month, may contribute to sleep disturbances [35]. During this period, patients with epilepsy who quit smoking may experience alterations in sleep architecture, increasing their likelihood of receiving a new sleep disorder diagnosis.

The extended time-stratified Cox regression analysis, which divided the 10-year follow-up period into five two-year intervals, revealed that the risk of sleep disorders remained elevated (1.4–2.2 times higher) during the first eight years following an epilepsy diagnosis but declined thereafter to a statistically nonsignificant level. The highest risk increase was observed during the zero-to-two-year period after diagnosis. In the subgroup analysis based on sex and age, differences were noted in the risk trajectory over the eight-year period. Among men and individuals aged younger than 60 years, the increased risk remained significant throughout the 8-year period. By contrast, among women and individuals aged 60 years or older, the increase in risk was not significant during the two- to four-year and six- to eight-year intervals. These differences may be related to physiological and psychosocial variations across sex and age groups, highlighting the need for further research to elucidate the underlying mechanisms.

The marked increase in sleep disorder risk during the first two years after an epilepsy diagnosis is likely associated with psychosocial stress following the diagnosis and the challenges of initial seizure management. Despite ongoing efforts to improve awareness, the social stigma surrounding epilepsy persists [38]. Additionally, the fear of unpredictable seizures, unemployment, and financial strain can aggravate stress levels in affected patients [39]. This psychological distress may be particularly pronounced during the first year after diagnosis [17]. The nature of epilepsy treatment may also contribute to the increased risk of sleep disorders during the early stages following diagnosis. Epilepsy management is largely empirical and requires a trial-and-error approach as treatment is tailored to individual seizure types and responses. This process can last months or years before optimal disease control is achieved [40, 41]. During the initial phase of treatment, identifying the appropriate combination and dosage of AEDs is crucial; however, dose adjustments and potential side effects may significantly alter sleep architecture. These factors likely contribute to the heightened vulnerability to sleep disorders among patients during the early post-diagnosis period.

From a clinical perspective, these findings suggest that early screening for sleep disorders should be incorporated into the routine evaluation of patients with newly diagnosed epilepsy. The early post-diagnosis period represents a critical window when patients experience the greatest psychosocial and neurophysiological vulnerability. Timely identification and management of sleep disturbances during this period may help improve seizure control, treatment adherence, and overall quality of life.

This study used a large-scale South Korean cohort to evaluate the risk of sleep disorders in patients with epilepsy and to identify the associated factors. Additionally, it examined changes in sleep disorder risk over time following diagnosis, helping to fill the gaps in the existing knowledge regarding the relationship between epilepsy and sleep disorders. However, some limitations should be acknowledged. First, detailed clinical information such as seizure frequency and patterns of anti-seizure medication (ASM) use was unavailable, which may have influenced the observed associations. Although major demographic and lifestyle factors were adjusted for, unmeasured clinical variables, including seizure severity and comorbid mood disorders, could also have contributed to the findings. Future studies incorporating detailed clinical and psychiatric data, as well as longitudinal seizure tracking, are warranted to clarify these relationships. Second, the reliance on ICD-10 codes for outcome identification may have led to underestimation of undiagnosed or subclinical sleep disorders, as routine sleep screening information was not accessible. Third, the relatively short mean follow-up period of about four years limits interpretation of the later time intervals, and the higher IRR observed among younger adults likely reflects their lower baseline prevalence of sleep disorders. Fourth, individuals younger than 20 years were not included in this analysis. Sleep disorders in adolescents with epilepsy are known to significantly affect quality of life, disease control, and cognitive function [42, 43]; therefore, future studies should consider age-specific characteristics and include younger populations. Finally, this study was conducted exclusively in a South Korean population, and cultural and healthcare system differences may affect the generalizability of the findings to other regions. Future research integrating seizure frequency data, detailed ASM information, and more diverse populations will be essential to enhance understanding of the relationship between epilepsy and sleep disorders across different settings.

## Conclusions

This study identified that patients with epilepsy were associated with a significantly higher risk of sleep disorders than the control group, with the risk being particularly pronounced in the early post-diagnosis period, as well as among men and younger individuals. Additionally, the risk of sleep disorders persisted for approximately eight years after diagnosis before declining to a statistically nonsignificant level. These findings highlight the importance of the early detection and management of sleep disorders, particularly in high-risk patients with epilepsy. Clinicians should examine sleep disturbances in these patients and implement appropriate intervention strategies to improve their overall quality of life.

## Supplementary Information

Supplementary Material 1.

## Data Availability

The datasets analyzed in this study can be obtained from the corresponding authors upon reasonable request.

## Abbreviations

AEDs: Antiepileptic drugs
aHR: Adjusted hazard ratio
BMI: Body mass index
CI: Confidence interval
FBS: Fasting blood sugar
ICD-10: International Classification of Diseases, 10th Revision
IR: Incidence rate
IRR: Incidence rate ratio
KCD-8: Korean Standard Classification of Diseases, 8th revision
KNHIS: Korean National Health Insurance Service
NHSP: National Health Screening Program
SD: Standard deviation
