# Supplementary material for: Time-dependent risk of sleep disorders in patients with epilepsy: a nationwide cohort study
Source: BMC Neurol. 2026 Jun 10;26:516. doi: 10.1186/s12883-026-05038-6 (PMC13474655; doi:10.1186/s12883-026-05038-6)
Supplement: Supplementary file 1 [file 12883_2026_5038_MOESM1_ESM.docx]

**Supplementary Material**

**Table S1.** Korean Standard Classification of Diseases 8th revision (KCD-8) codes for diseases.

| Disease | | KCD-8 codes |
| --- | --- | --- |
| Epilepsy | Generalized epilepsy | G40.2, G40.3, G40.4, G40.5, G40.6 |
|  | Focal epilepsy | G40.0, G40.1, G40.7 |
|  | Other epilepsy | G40.8, G40.9 |
|  | Status epilepticus | G41 |
| Sleep disorder | | F51, G47 |
